# Transcriptome analysis reveals that PRV XJ delgE/gI/TK protects against intestinal damage in nose-dropping-infected mice by regulating ECM-ITGA/ITGB-P-FAK

**DOI:** 10.1128/spectrum.01828-24

**Published:** 2024-11-29

**Authors:** Tong Xu, Yang Zhang, Qian Tao, Lei Xu, Si-Yuan Lai, Yan-Ru Ai, Jian-Bo Huang, Ben-Lu Yang, Ling Zhu, Zhi-Wen Xu

**Affiliations:** 1 College of Veterinary Medicine, Sichuan Agricultural University, Chengdu, China; 2 College Of Animal Science and Technology of Jiangxi Agricultural University, Nanchang, China; 3 College of Veterinary Medicine Sichuan Key Laboratory of Animal Epidemic Disease and Human Health, Sichuan Agricultural University, Chengdu, China; Universidade Federal do Rio de Janeiro, Rio de Janeiro, Brazil

**Keywords:** pseudorabies virus, focal adhesion kinase, transcriptome, drip-nose PRV-infection

## Abstract

Pseudorabies virus (PRV) is an ideal model for mechanistic investigations into α-herpesvirus. The neurotropism and latent infection of PRV have been extensively studied. Apart from neurological symptoms, diarrhea caused by PRV infection is also an essential cause of mortality in newborn and weaned piglets. However, little research has been done on PRV invasion of the gut. To fill this gap, a nasal drip PRV-infection mouse model was developed, consisting of three groups: the challenged group (Group A), the immunization-challenged group (Group B), and a mock group (Group C). The results showed that immunization with PRV XJ delgE/gI/TK successfully prevented intestinal damage caused by PRV drop-nose infection. Subsequently, intestines were collected for transcriptional analysis. Differentially expressed genes analysis revealed that PRV XJ delgE/gI/TK was effective in reducing the organismal intestinal transcriptional activity caused by PRV. The Group A vs Group C and Group A vs Group B had similar Kyoto Encyclopedia of Genes and Genomes (KEGG)-enriched signaling pathways and the differentially expressed genes were primarily enriched in pathways, such as cell adhesion molecules, focal adhesion kinase, and actin cytoskeleton regulation. Notably, transcriptome analysis indicated that genes associated with the focal adhesion kinase (FAK) signaling pathway (ECM-ITGA/ITGB-p-FAK) were significantly more highly expressed in Group A than in Group B and Group C. The results of quantitative real-time PCR (RT-qPCR) and western blotting were consistent with KEGG analysis. Therefore, we hypothesized that PRV promotes self-infection through activation of the ECM-ITGA/ITGB-p-FAK signaling pathway and that PRV XJ delgE/gI/TK immunization could attenuate the intestinal damage caused by PRV by inhibiting the activation of this pathway.

**IMPORTANCE:**

Pseudorabies virus (PRV) poses a significant threat to the swine industry and public health due to its ability to infect multiple species, including humans, leading to substantial economic losses and potential health risks. This study addresses a critical gap in understanding the impact of PRV infection on the gut, which has been less explored compared to its neurological effects. By developing a drip-nose PRV-infection mouse model, the research indicated that PRV might promote self-infection through activation of the ECM-ITGA/ITGB-p-FAK signaling pathway, and PRV XJ delgE/gI/TK immunization effectively prevents intestinal damage by significantly reducing the expression of genes in the ECM-ITGA/ITGB-p-FAK signaling pathway. The research has important implications for the swine industry and public health by contributing to the development of better vaccines and treatments, ultimately helping to control PRV and prevent its cross-species transmission.

## INTRODUCTION

Pseudorabies (PR) is an acute and severe infectious disease of swine worldwide caused by the Pseudorabies virus (PRV), posing a significant threat to the pork industry (1). PR was first described in the United States as early as 1813 and subsequently spread to many countries around the world, with the first recorded case in China occurring in 1948 (2). After that, PR broke out in many areas of China, causing serious economic losses to the pig industry (3). The gE-negative vaccine (Bartha-K61) derived from Hungary was introduced into China in the 1970s, and PR was well controlled in China thereafter (4–6). Since late 2011, PR re-emerged in many Bartha K61-vaccinated swine herds and spread quickly across various provinces in China (7). Sequence analysis showed that the newly emerged PRV strains clustered in different branches from those previously classical strains in China (8, 9). Newly emerging PRV strains are often named PRV variants to distinguish them from pre-2011 epidemic strains (classical strains). Moreover, the Bartha-K61 vaccine cannot provide complete protection against emerging PRV variants (7, 10, 11).

Besides its natural host, pigs, PRV can infect many other species, including ruminants, carnivores, and rodents (1, 12). Recently, more than 20 PRV infection cases in humans have been reported (3, 13), and all these patients infected with PRV variants had close contact with pigs, suggesting that pigs may be a source of transmission of PRV infection in humans (14). Therefore, researchers should pay more attention to the cross-species transmission of PRV, as well as its pathogenicity. After infection with PRV, pigs at different stages display different symptoms along with high mortality (7). For instance, neonatal piglets exhibit diarrhea, fever, and neurological symptoms; weaned piglets show vomiting abdomen, diarrhea outside, and neurological symptoms; nursery pigs usually display fever, coughing, and respiratory disease (15).

PRV is a member of the subfamily α-herpesviruses and the genus Varicella, with a double-stranded genomic DNA of approximately 143 kb (16, 17). PRV is an ideal model for mechanistic investigations into α-herpesvirus (17, 18). Neurophilicity and latent infection of PRV have been extensively studied (19, 20). A new *in vitro* model of PRV latency has been established, showing that the viral UL54 gene plays a crucial role in the reactivation process, providing a streamlined method to study these mechanisms (21). The UL37 gene product of the Pseudorabies virus is essential for secondary envelopment, facilitating the addition of tegument proteins needed for complete viral assembly (22). Recent research utilizing biotin-based proximity labeling has identified host proteins involved in the early stages of PRV and HSV-1 infections, notably highlighting zyxin as a novel inhibitory factor against viral entry and spread (23). The results of one study confirmed that mutations in these envelope proteins (especially gD) greatly enhance the ability of PRV to attach and penetrate (24). PRV has been found to inhibit progesterone-induced inactivation of TRPML1 to facilitate viral entry, shedding light on the virus’s ability to impair female fertility by disrupting key reproductive hormonal pathways (25). However, little research has been done on PRV invasion of the gut. Apart from neurological symptoms, diarrhea caused by PRV infection is also an essential cause of mortality in newborn and weaned piglets.

Focal adhesion kinase (FAK) is an important non-receptor protein tyrosine kinase that plays an important role in intercellular adhesion and adhesion between cells and the ECM. Additionally, it is closely related to embryonic development, cell cycle regulation, and angiogenesis. Upon activation, FAK undergoes a conformational change that leads to the exposure of the catalytic domain, while autophosphorylation and phosphorylated FAK (p-FAK) formation occur (26). Activated FAK is involved in a wide range of biological processes through multiple pathways.

In this study, the PRV mouse nasal drip infection model was used to screen for differentially expressed genes in the gut among the immunization-challenged group, challenged group, and mock groups. The aim of this study was to investigate the mechanisms by which PRV causes intestinal pathogenicity and by which PRV delgE/gI/TK attenuates intestinal damage caused by wild strains. This study has important implications for understanding the mechanisms of PRV damage to the gut and for the development of anti-PRV drugs.

## MATERIALS AND METHODS

### Virus and cells

PRV-XJ (Genbank accession No. MW893682.1) strain was isolated from the brain of a dead piglet that had been vaccinated from a PRV-infected pig farm in Sichuan province, China, and was preserved by the College of Veterinary Medicine, Sichuan Agricultural University (Chengdu, China). C57BL/6J mice were purchased from Beijing Huafukang Biotechnology Co., LTD. The mice were kept at room temperature (23°C ± 1.5°C) with free access to food and water.

### Determination of LD_50_ in a mouse model

The PRV XJ strain was diluted 10-fold (10^1^–10^7^) in serum-free Dulbecco's Modified Eagle Medium (DMEM). Forty C57 BL/6 mice were randomly divided into seven experimental groups (groups I–VII) and one mock group (group VIII), with five mice in each group. A 100 µL dose of PRV XJ at a dilution of 10^1^–10^7^ was given by nasal drip to each of the mice in groups I–VII, respectively, and the mock group (group H) was injected with the same dose of DMEM. Mouse morbidity and mortality were observed daily for 14 days. The LD_50_ of the virus was calculated by the Reed-Muench method.

### Construction of PRV-infected mouse model

**Fig 1:**
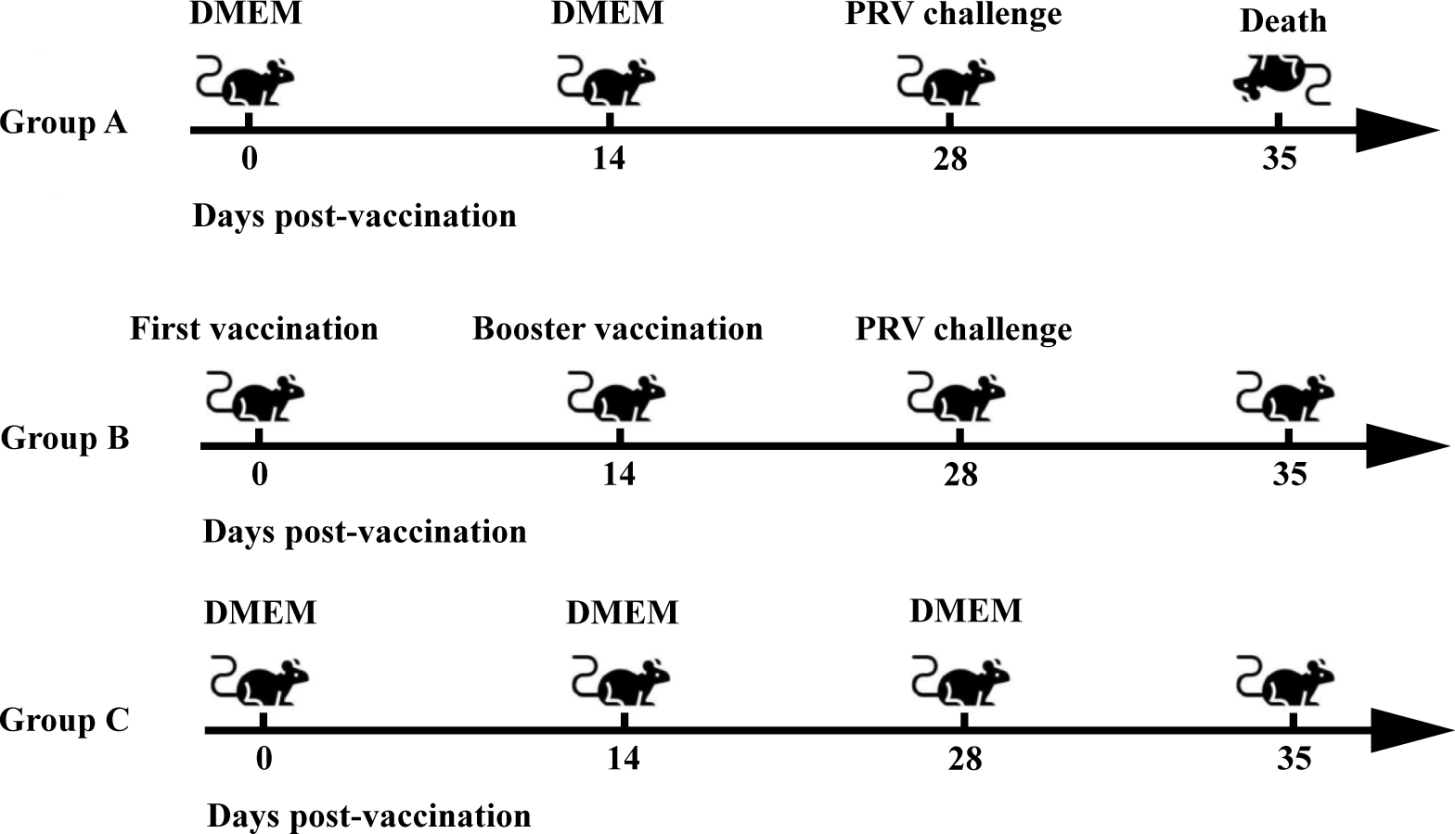
Construction of PRV-infected mouse model. Group A: the challenged group; Group B: immunization-challenged group; Group C: a mock group.

Fifteen 4-week-old female C57 BL/6 mice were randomly divided into three groups, the challenged group (Group A), the immunization-challenged group (Group B), and a mock group (Group C), with five mice in each group. The grouping and treatment of mice are shown in Fig. 1. Group B: 10^6^ TCID_50_ of PRV XJ Del gE/gI/TK viral solution was administered by nasal drip. Then, these groups were booster-immunized with the same dose 14 days postvaccination. Groups A and C were inoculated with the same amount of serum-free DMEM at the time of the first immunization and at the time of the booster immunization in the same manner. On day 28 after the initial immunization, mice in groups A and B were challenged with 100 µL of 10 × LD_50_ of PRV XJ by nasal drip. Group C was injected with the same dose of serum-free DMEM in the same way. Afterward, each group of mice was monitored daily. Dying mice were humanely euthanized with an overdose of pentobarbitalum natricum (40 mg/kg). Seven days later, all surviving mice were humanely euthanized using the same method. Brain, lung, and intestinal tissues were collected and stored in liquid nitrogen or 4% paraformaldehyde.

A quantitative real-time PCR assay targeting the PRV gE gene, previously reported in our laboratory was used to determine the viral load in the main organs (including brain, lungs, and intestines) of each group of mice (19). The specific primers targeting gE are summarized in Table 1. The viral DNA copy number was expressed as log10 copies per gram of tissue sample. The main organs of each group of mice were sent to Chengdu Lilai Biological Co., Ltd. for hematoxylin–eosin (HE) staining and histopathological observation after being fixed with 4% paraformaldehyde.

**TABLE 1:** Primers for qRT-PCR

Primer name	Primer sequence (5’→3’）	
	Forward primer	Reverse primer
PRV-gE	CTTCCACTCGCAGCTCTTCT	TAGATGCAGGGCTCGTACAC
β-actin	GCTGTGCTATGTTGCTCTAG	CGCTCGTTGCCAATAGTG
Caveolin1	CCCATTCCTGCTCTCTCTTTT	GTAGGCAGTTGAGGTTGTTGG
Jun	CCTTCTACGACGATGCCCT	TCGGCCAGGTTCAAGGTCA
KIf6	GTTAGTGGGTGGGATGGTTG	TACTCCTCACACACAAAAC
Muc13	CCCTAATCCCTACGCAAACCA	GCCCATTTCTCCTTGTCCTT
Abhd2	GACCACGTTAAGAAACCCCAG	CCTCATGCAGCTCTCTTCCTC
Apob	GCATTCCATTGTTGTCCCTCT	TTGCTTCATTATAGGAGGTGG
ECM2	TGGAAGAAGGGAGGAAGAGGAG	GGAATTACCCGGGAGGACATTC
ITGA1	TCGTTCGCCCCTGTACAAGA	GGGCCAATGTCCATCCTCTT
ITGB1	GTGCAATTGTCAAAGCCATG	TACATTCACAGTGCCTCCCA
PAK3	GATGACAATGAACCTCCGCC	ATTTGGTGCAGCTGGTGAAG
PI3K	AACAATGCCAAACCCAGGAG	CGGTACTCAGCTGCCTGCTT

### RNA extraction library construction and sequencing and RNA-seq data analyses

As described in the section Construction of PRV-infected mouse model, after the challenge, moribund mice or those still alive after 7 days were humanely euthanized with an overdose of pentobarbitalum natricum (40  mg/kg), and the ileum was collected and immediately stored in liquid nitrogen for subsequent transcriptome sequencing. Total RNA was extracted using Trizol reagent (thermofisher, 15596018) following the manufacturer’s procedure. The total RNA quantity and purity were analyzed with Bioanalyzer 2100 and RNA 6000 Nano LabChip Kit (Agilent, CA, USA, 5067-1511), and high-quality RNA samples with a RIN number >7.0 were used to construct the sequencing library. Subsequently, all RNA samples were sent to LC-Bio Technology CO., Ltd. (Hangzhou, China) for sequencing.

Reads containing adapters, poly A and poly G, more than 5% unknown nucleotides (N), and low-quality reads (Q value ≤ 20) were removed. The sequence quality was verified using FastQC including the Q20, Q30, and GC-content of the clean data. We aligned reads of all samples to the mus musculus C57BL/6J reference genome using the HISAT2 package. The mapped reads of each sample were assembled using StringTie with default parameters. Then, all transcriptomes from all samples were merged to reconstruct a comprehensive transcriptome using gffcompare software. After the final transcriptome was generated, StringTie and Ballgown were used to estimate the expression levels of all transcripts and perform expression abundance for mRNAs by calculating fragment per kilobase of transcript per million mapped reads (FPKM) value.

Differential gene expression analysis was performed by DESeq2 software between two different groups. The genes with the parameter of false discovery rate below 0.05 and |log2(FoldChange)| ≥ 2 were considered differentially expressed genes. The Pearson correlation coefficient between two replicas was calculated to evaluate repeatability between samples. Principal component analysis was performed with the princomp function of R in this experience. Differentially expressed genes were then subjected to enrichment analysis of Gene Ontology (GO) functions and Kyoto Encyclopedia of Genes and Genomes (KEGG) pathways using cluster Profiler software (v.3.8.1).

### qRT-PCR analyses of gene expression

In order to verify the accuracy of the transcriptome sequencing results, six differential genes were randomly selected, and six pairs of specific primers and internal reference gene primers were designed based on published mRNA sequences in NCBI, and the results were analyzed by the 2^-ΔΔCt^ method. In addition, primers for four differential genes related to the FAK signaling pathway were designed. The primer sequences are shown in Table 1.

### Western blotting analysis

For protein analysis, the intestinal tissues were homogenized in RIPA buffer containing protease inhibitors. Western blotting was performed as previously described in our laboratory’s published protocols (20). Primary antibodies included p-FAK (CST, USA), ITGB (CST, USA), ITGA (CST, USA), ECM2 (Proteintech, China), and β-Actin (Abcam, USA).

### Statistical analysis

Data processing in this study was performed using GraphPad Prism 9.5.1 software, and statistical differences between the two groups were analyzed by a t-test (Student’s two-tailed unpaired t-test), and differences between multiple groups were comparatively analyzed using one-way analysis of variance. A *P*-value < 0.05 was considered statistically significant, and the results were expressed as mean ± SD, *P < 0.05, **P < 0.01, ***P < 0.001, ****P < 0.0001.

## RESULTS

### The LD_50_ of mouse infection model

PRV XJ was inoculated into each group of mice by nasal drip, and a mock group was set up at the same time. The mice were continuously observed for death within 14 days after the challenge, and the results are shown in Table 2. All mice in groups I–IV died within 7 days, while mice in groups V and VI experienced partial mortality, and mice in group VII did not die. All dead mice showed clinical symptoms such as reduced appetite and itching and scratching of the ears in the pre-mortem period. The LD_50_ of the virus administered by nasal drip was calculated as 10^-5.25^/0.1 mL by the Reed-Muench method.

**TABLE 2:** LD_50_ in the mouse by intranasally inoculated

Dilution	Number	Death	Survival	Accumulated number of deaths	Accumulated number of survival	Total	Fatality rate (%)
10^−1^	5	5	0	24	0	24	100
10^−2^	5	5	0	19	0	19	100
10^−3^	5	5	0	14	0	14	100
10^−4^	5	5	0	9	0	9	100
10^−5^	5	3	2	4	2	6	60
10^−6^	5	1	4	1	6	7	20
10^−7^	5	0	5	0	11	11	0

### PRV del gE/gI/TK protects infected mice from death

**Fig 2:**
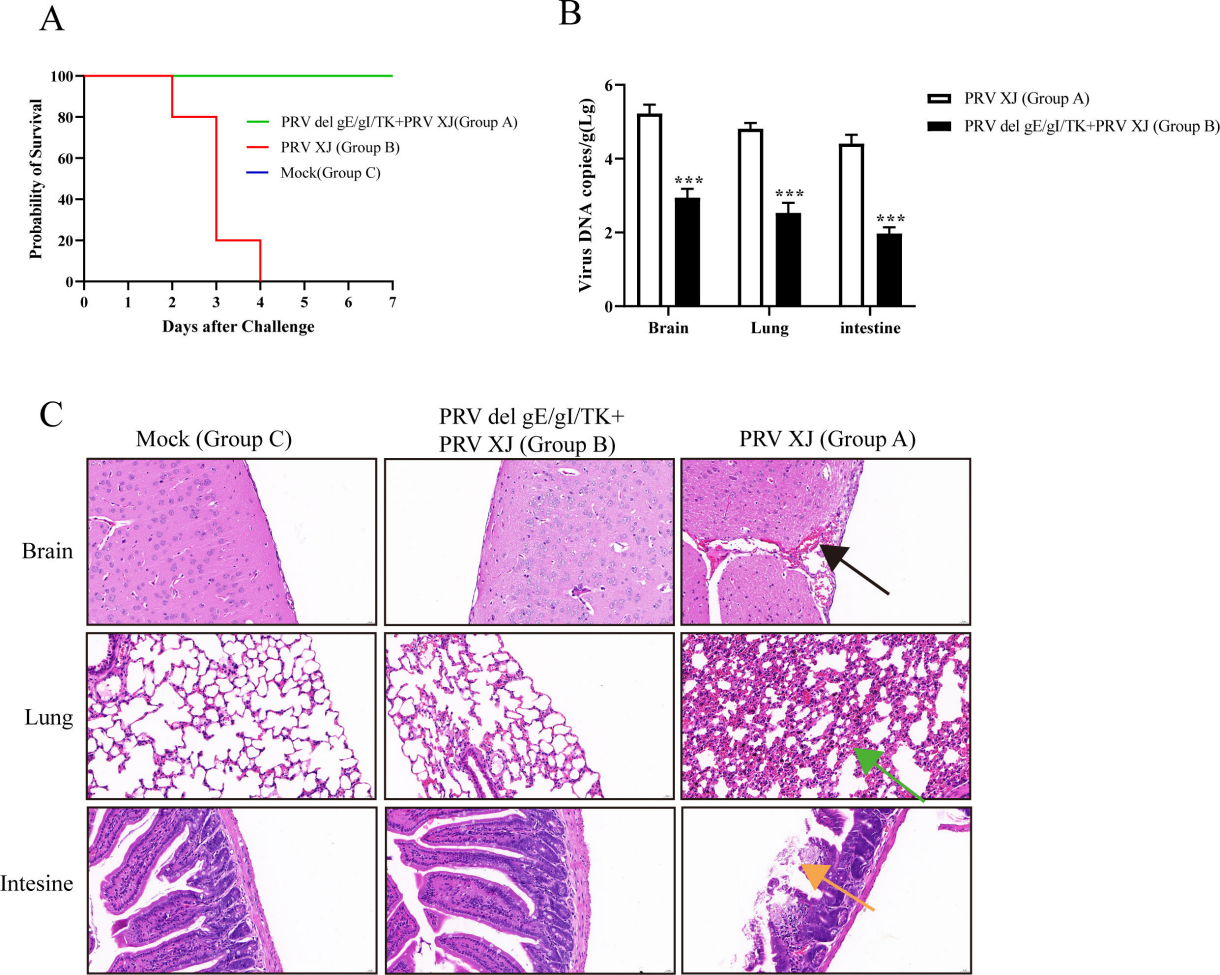
Validation of PRV infection model in mice. (**A**) Survival curves of PRV-infected mice. (**B**) Viral load measurements of brain, lung, and intestinal tissues of mice. (**C**) Pathologic observations of H&E staining of brain, lung, and intestines. Black arrows: localized congestion and widening of meninges; green arrows: localized widening of alveolar walls and slight siltation of alveolar wall capillaries; orange arrows: apical lysis of the mucosal layer and detachment of epithelial cells.

Based on LD_50_ results, a dose of 10 × LD_50_ was selected to test the challenged group (Group A) and the immunization-challenged group (Group B), while a mock group (Group C) was set up at the same time. Survival and clinical signs of each group of mice were observed daily after the challenge. Group A showed obvious clinical symptoms and started to die on the second day, and all of them died within 4 days. In contrast, mice in Groups B and C did not show any clinical symptoms and did not die after the challenge (Fig. 2A). As depicted in Fig. 2, the viral loads in the brain, lungs, and intestines of the mice in Group B were significantly lower than those of the mice in Group A (Fig. 2B). In addition, the results of HE staining (Fig. 2C) demonstrated that the brain, lungs, and intestines of mice in Group A showed the typical pathological changes after PRV infection, which were mainly characterized by widening of meningeal congestion, thickening of the alveolar walls, and slight bruising, as well as dissolution and detachment of the apical mucous layer of the intestines. Nevertheless, no pathological changes were observed in both groups B and C. These findings indicate that mice in both Groups A and B were successfully infected with PRV, that infection with PRV XJ is lethal to mice, and that PRV del gE/gI/TK provides effective protection to mice. Intestinal tissue samples from all mice in the three groups were further used for transcriptomic analysis.

### RNA sample quality testing and sequencing data quality assessment

Tissue total RNA was checked for quality using the 4200 TapeStation automated electrophoresis system. The RNA quality check peaks mainly show ribosomal RNA signals, and RNA integrity is mainly judged by the distribution of ribosomal RNA fragments. The more obvious ribosomal RNA peaks indicated better RNA integrity. As displayed in Fig. S1, the ribosomal RNA peaks were prominent in all samples, indicating high RNA integrity. More detailed results of the quality check of RNA are shown in Table 3. The OD260/280 values were all greater than 1.9, indicating no protein contamination, and the OD260/230 values were all greater than 2, indicating no chemical reagent residues. These data indicate that the RNA quality meets the requirements for further library construction and sequencing.

**TABLE 3:** Summary table of RNA test results

Sample name	Concentration ng/μL	Total amount μg	OD260/280	OD260/230
Group A1	0.87	24.47	2.02	2.20
Group A2	0.88	24.51	2.02	2.20
Group A3	0.88	24.57	2.01	2.19
Group A4	0.88	25.20	2.02	2.22
Group A5	0.90	25.23	2.02	2.22
Group B1	0.69	19.39	2.00	2.06
Group B2	0.69	19.23	2.02	2.19
Group B3	0.68	19.03	2.02	2.17
Group B4	0.68	19.07	2.01	2.20
Group B5	0.68	19.10	2.02	2.22
Group C1	1.74	48.78	2.03	2.26
Group C2	1.78	49.72	2.03	2.26
Group C3	1.72	48.20	2.03	2.24
Group C4	1.68	47.15	2.02	2.24
Group C5	1.67	46.89	2.02	2.22

The quality of the sample sequencing results was assessed as shown in Table 4. The Clean bases obtained by filtering out the reads with connectors and low quality totaled 82.35G. The Q20 values of all samples exceeded 99%, the Q30 values exceeded 96%, and the GC content was around 50%, indicating that none of the samples showed AT/GC separation, which demonstrates that the sequencing data were of high quality and could meet the requirements for subsequent data analysis.

**TABLE 4:** Summary table of RNA-seq sequencing data quality evaluation

Sample name	Raw_reads	Clean_reads	Clean_bases	Error rate (%)	Q20 (%)	Q30 (%)	GC content (%)
Group A1	38182920	36784216	5.52G	3.66	99.96	96.83	48
Group A2	38062088	36561984	5.48G	3.94	99.86	96.59	48.50
Group A3	38862344	37329910	5.60G	3.94	99.88	96.93	48
Group A4	39172392	37619724	5.64G	3.96	99.88	96.78	48
Group A5	37384836	35925814	5.39G	3.9	99.86	97.32	48
Group B1	37711952	34990678	5.25G	7.22	99.95	97.10	97.10
Group B2	47388348	43944748	6.59G	7.27	99.94	96.63	47.50
Group B3	38604058	36199086	5.43G	6.23	99.96	97.20	47.50
Group B4	40063580	37225052	5.58G	7.09	99.95	96.95	47.50
Group B5	37507406	33446440	5.02G	10.83	99.95	96.84	47.50
Group C1	38288362	33732874	5.06G	11.9	99.83	99.83	47.50
Group C2	39149490	34769140	5.22G	11.19	99.83	99.83	46.50
Group C3	38451776	35270790	5.29G	8.27	99.83	96.81	96.81
Group C4	40331106	37732942	5.66G	6.44	99.96	97.22	97.22
Group C5	40487144	37435518	5.62G	7.54	99.88	99.88	99.88

### Analysis of differentially expressed genes

**Fig 3:**
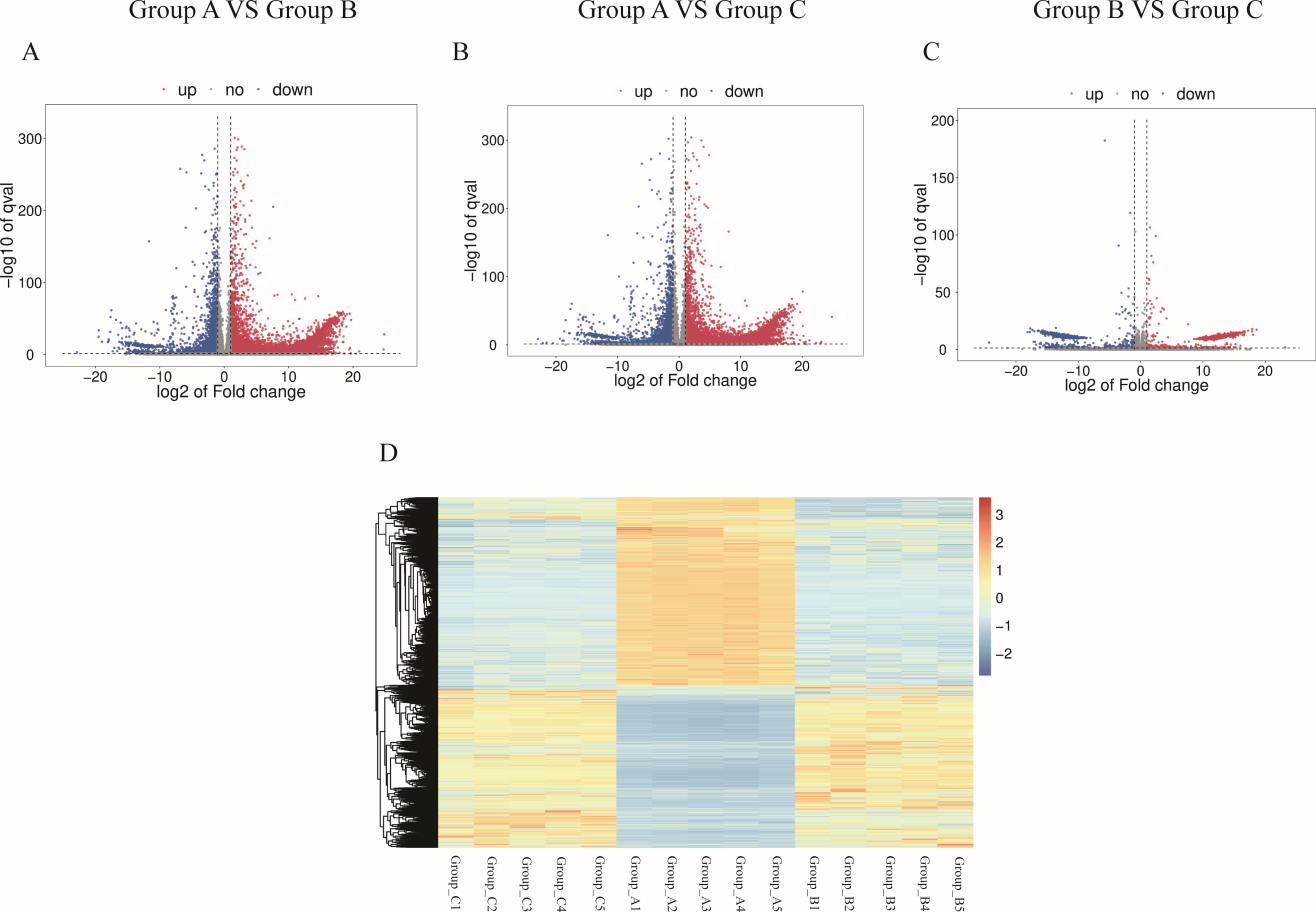
Differential expression gene analysis. (**A**) Differential gene expression volcano plot for the challenged Group (Group A) vs immunization-challenged group (Group B). (**B**) Differential gene expression volcano plot for the challenged group (Group A) vs mock group (Group C). (**C**) Differential gene expression volcano plot for Group B vs Group C. (**D**) Cluster heatmap of differential expression genes based on hierarchical cluster analysis.

In order to screen the differentially expressed genes in mice of different treatment groups, the challenged group (Group A), immunization-challenged group (Group B), and a mock group (Group C) were compared pairwise (Fig. 3). Briefly, there were 3,646 up-regulated genes and 2,703 down-regulated genes in group A compared to group C (Fig. 3A). There were 3,858 up-regulated genes and 2,663 down-regulated genes in group A compared to group B (Fig. 3B). There were 93 up-regulated genes and 216 down-regulated genes in group B compared to group C (Fig. 3C).

Based on the similarity of the gene expression profiles of the samples, the genes were clustered and analyzed, and the FPKM values of the differentially expressed genes were used as the basis for the clustering analysis, and the clustering heat map was drawn. The results indicated that the trend of differentially expressed genes between different samples in the same group was the same (Fig. 3D).

### GO enrichment analysis of differentially expressed genes

GO is an internationally standardized classification system for gene function, and there are three ontologies in GO, which describe the molecular function (MF), cellular component (CC), and biological process (BP) of genes. The basic unit of GO is a term, and each term corresponds to an attribute. top25, top15, and top10 terms from MF, CC, and BP were selected to draw bar charts for presentation (Table S1).

**Fig 4:**
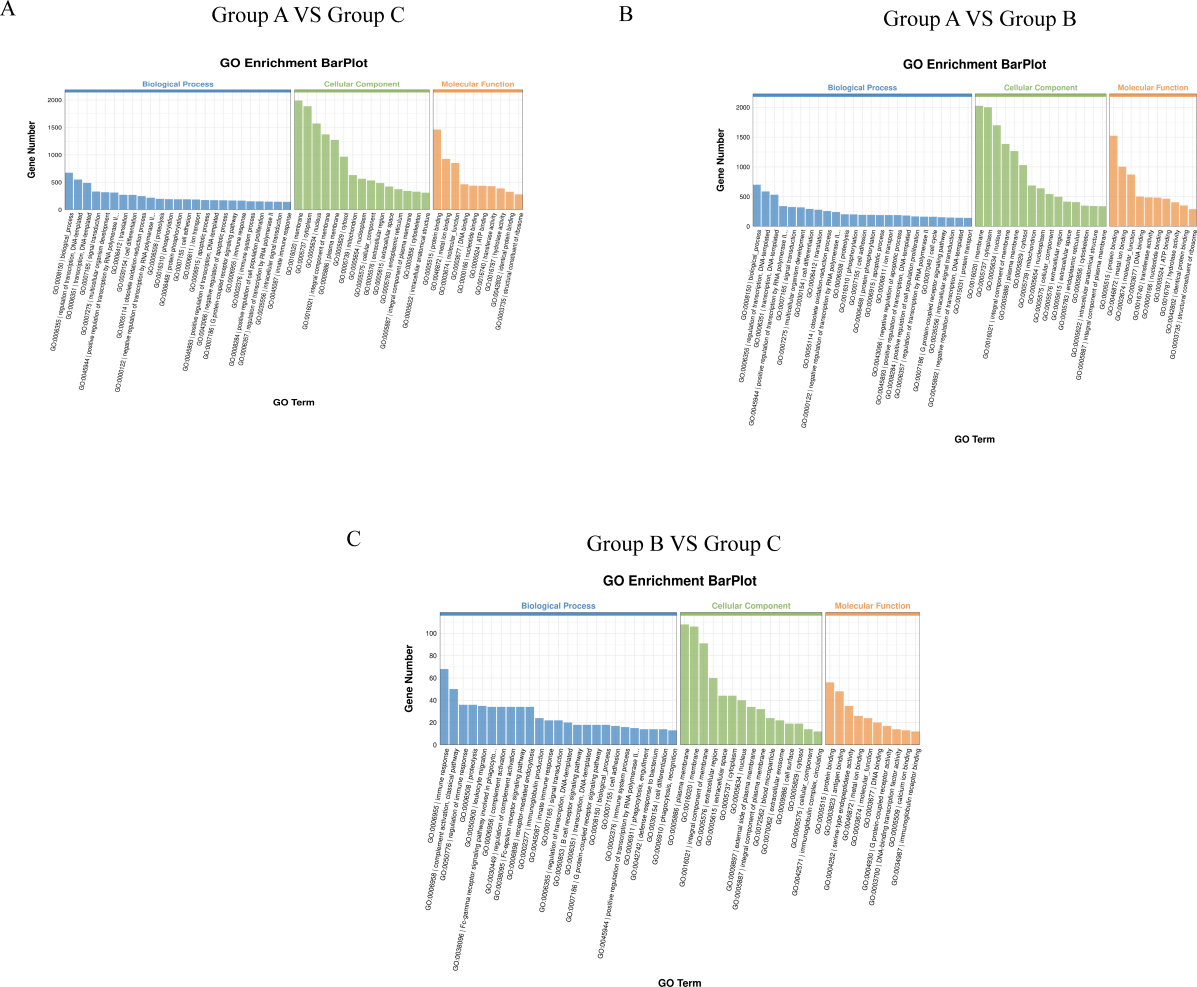
GO function enrichment analysis of differentially expressed genes. (**A**) Enriched GO terms in Group A vs Group C. (**B**) Enriched GO terms in Group A vs Group B. (**C**) Enriched GO terms in Group B vs Group C. Group A, Group B, and Group C represent the challenged group, the immunization-challenged group, and the mock group.

As shown in Fig. 4A and B, group A vs group C comparator group and group A vs group B comparator group have similar enriched terms. Enrichment terms for BP, CC, and MF are summarized in Table S1. In the comparison between Group B and Group C, the GO enrichment terms were significantly different from those in the other two comparison groups (Fig. 4C), with detailed results summarized in Table S1.

### Enrichment analysis of the KEGG pathway for differentially expressed genes

**Fig 5:**
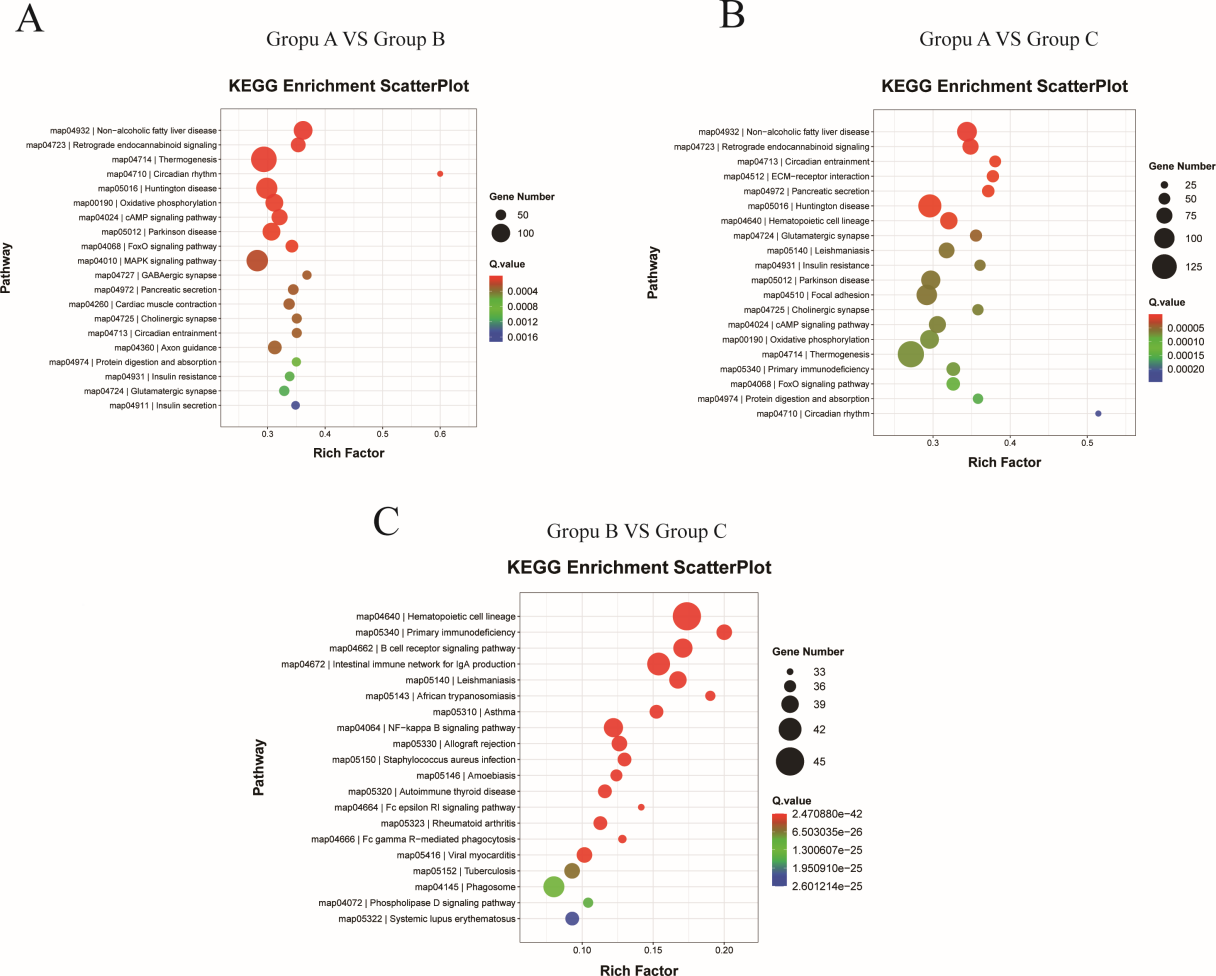
KEGG pathway enrichment analysis. (**A**) Scatterplot of the KEGG pathway enriched by differential expression genes for Group A vs Group B. (**B**) Scatterplot of the KEGG pathway enriched by differential expression genes for Group A vs Group C. (**C**) Scatterplot of the KEGG pathway enriched by differential expression genes for the (Group B) vs the mock group (Group C). Group A, Group B, and Group C represent the challenged group, the immunization-challenged group, and the mock group.

KEGG signaling pathway analysis of differentially expressed genes was performed to identify the pathways in which differentially expressed genes may be involved after viral infection of host cells. In order to investigate the potential molecular mechanisms that influence intestinal integrity after PRV infection, the 20 most significant KEGG pathways were selected from the KEGG enrichment results (Table S2). As shown in Fig. 5, Group A vs Group C and Group A vs Group B had similar KEGG-enriched signaling pathways, and significant changes were produced in several signaling pathways, such as cell adhesion molecules, adhesion plaques, and actin cytoskeleton regulation. The results of Group B vs Group C are shown in Fig. 5, the different signaling pathways were smaller. The top 20 KEGG pathways significantly enriched in Group B vs Group C were mainly enriched in immune-related pathways, such as the B-cell receptor signaling pathway, gut immune network generating IgA pathway, and NF-κB signaling pathway.

### qRT-PCR and western blotting analyses of gene expression

**Fig 6:**
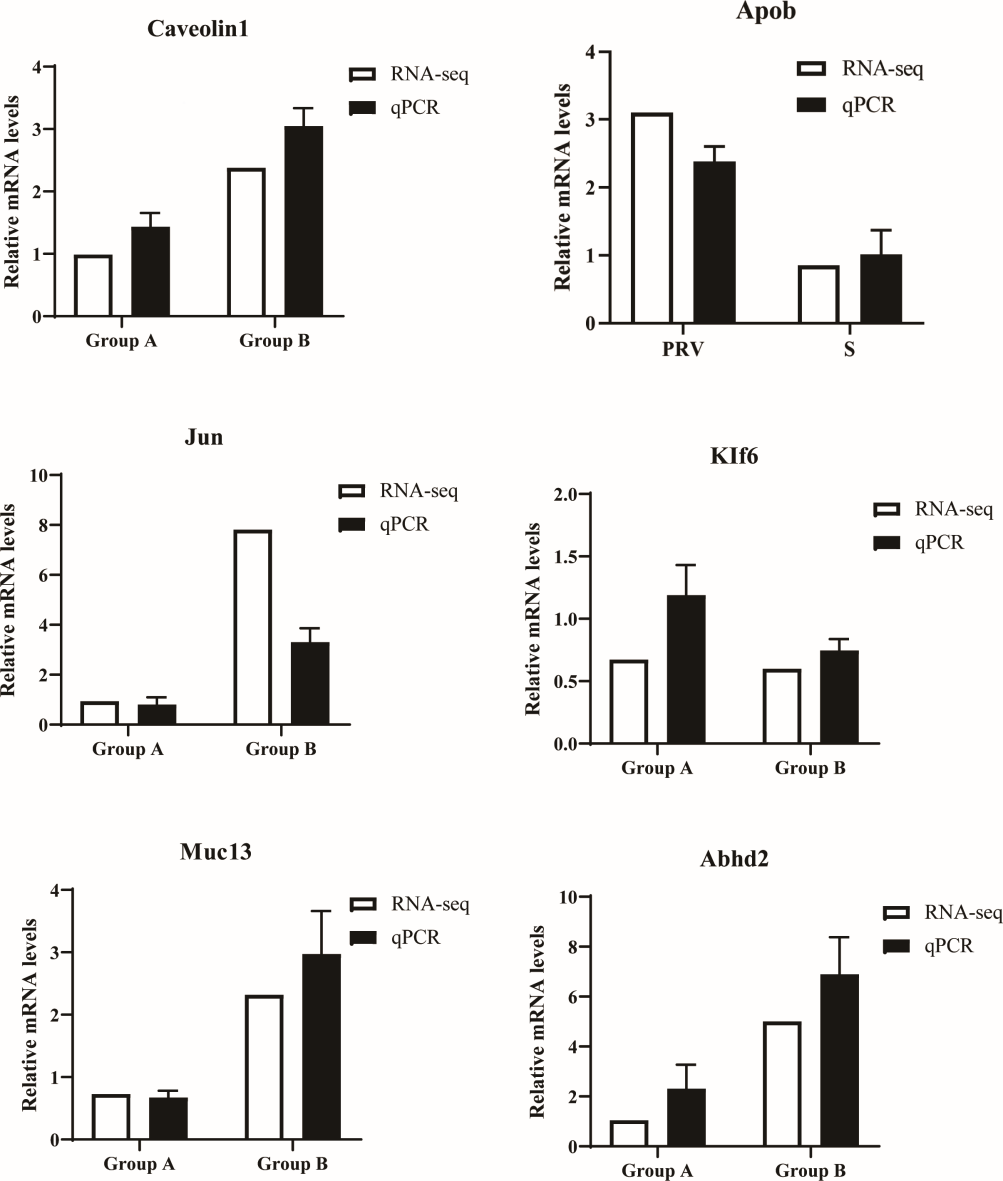
RT-qPCR validation of differential expression genes for the mRNA levels.

In order to verify the accuracy of the RNA-seq results, six genes were randomly selected for validation. The results are shown in Fig. 6. The relative gene expression results from RT-qPCR and transcriptome sequencing showed consistent trends in gene up-regulation or down-regulation, demonstrating that the transcriptome data are accurate and reliable.

**Fig 7:**
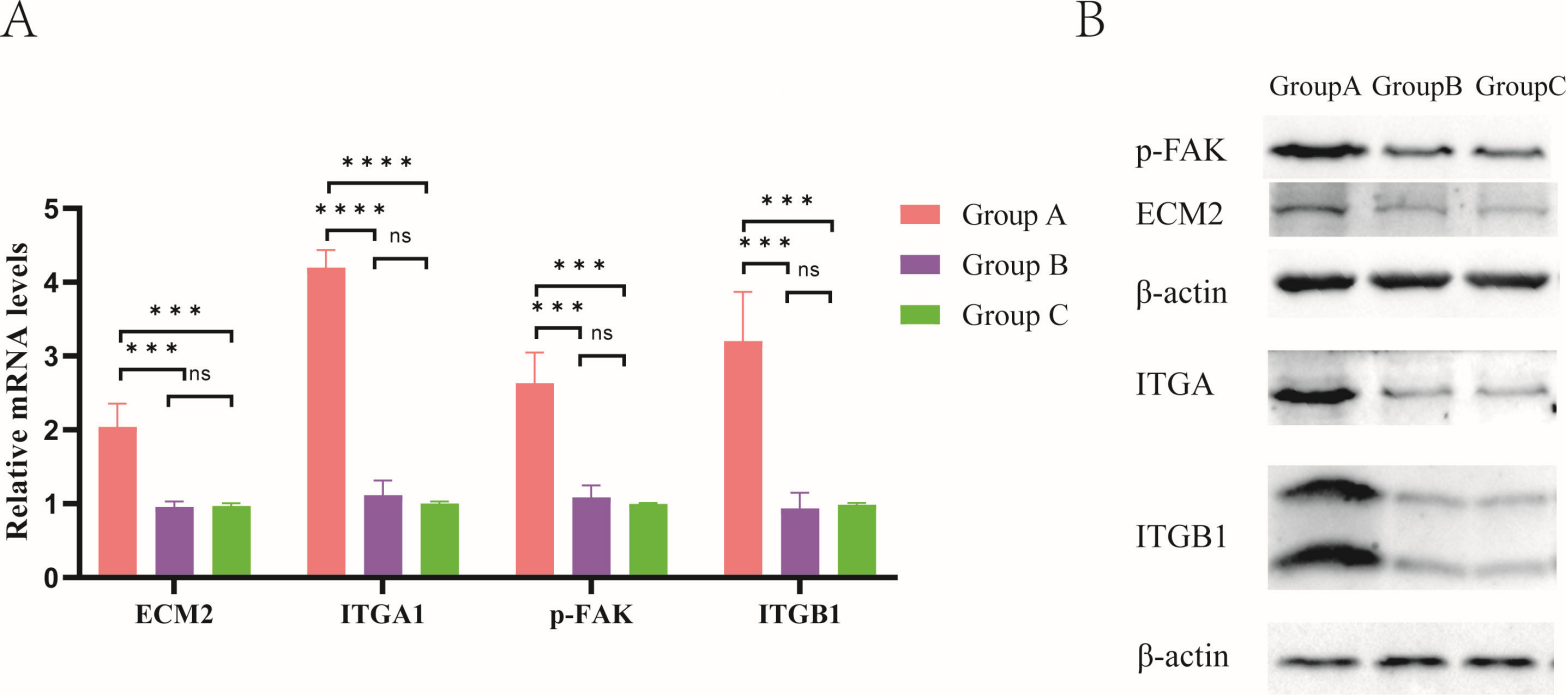
Changes in the protein expression levels of ECM2, ITGA1, p-FAK, and ITGB1. (**A**) The relative mRNA expression among the challenged group, immunization-challenged group, and the mock group. (**B**) Western blotting was used to assess the expression levels.

Notably, we found that genes associated with the FAK signaling pathway (ECM-ITGA/ITGB-p-FAK) were significantly more highly expressed in Group A than in Group B and Group C. Pathogenic microorganisms are often involved in manipulating FA thereby modulating the host cell actin backbone to facilitate their invasion. Therefore, we verified the expression of genes related to the FAK signaling pathway in PRV-infected mice by qPCR and western blotting (Fig. 7). The results showed that both the mRNA and protein levels of ECM, ITGA/ITGB, and p-FAK were significantly increased in the challenged group (Group A), whereas there was no difference between the immunization-challenged group (Group B) and the mock group (Group C) (Fig. 7).

## DISCUSSION

PRV consistently induces symptoms such as fever, respiratory distress, generalized neurological signs, and diarrhea in piglets (3, 11). Extensive studies have focused on the neurological and respiratory impacts of PRV (27). However, there are few reports on the effects of PRV on the intestinal aspects. The development and evaluation of PRV vaccines have focused on the observation of neurologic and respiratory injuries at the expense of intestinal injuries. In recent years, more and more studies on the brain-gut axis have shown that the gut and brain are inextricably linked (28, 29). Therefore, it is necessary to study the effects of PRV on the intestine and the protection of the intestine by vaccines, which will insight into directions and ideas for the subsequent studies on the pathogenesis of PRV and the development of vaccines.

In this study, we established a mouse model of drip-nose infection with PRV and analyzed the transcriptome of the mouse intestine. The intestinal transcriptomics of PRV-infected mice were analyzed to predict the potential pathway mechanisms of PRV infection. A pairwise comparison of the transcriptome results revealed that 3,646 transcripts were up-regulated and 2,703 transcripts were down-regulated in the PRV-infected group (Group A) compared to the mock group (Group C). In the differentially expressed genes, 3,858 transcripts were up-regulated and 2,663 transcripts were down-regulated in the PRV-infected group (Group A) compared to the immunization-challenged group (Group B). In the immunization-challenged group (Group B), 93 transcripts were up-regulated and 216 transcripts were down-regulated compared to the blank group. This indicates that PRV XJ delgE/gI/TK immunization significantly suppressed the transcriptional activity of PRV. To determine the potential mechanism of action on intestinal integrity after PRV infection, we focused on the signaling pathways related to intestinal integrity in the KEGG results. By analyzing the KEGG results of the vaccine group and the blank group, the signaling pathways related to intestinal integrity were hardly significantly altered. The top 20 KEGG-enriched signaling pathways, on the other hand, mainly focused on intestinal immunity-related, such as the B-cell receptor signaling pathway, the IgA pathway produced by the intestinal immune network, and the NF-κB signaling pathway. It showed that PRV XJ delgE/gI/TK immunization activated the intestinal immune system to defend against the attack of PRV wild strains. The results of the challenged group (Group A) showed that several signaling pathways related to intestinal integrity were significantly altered after PRV infection, such as cell adhesion molecules, FAK, and actin cytoskeleton regulation. FAK is involved in a wide range of biological processes through multiple pathways (30–32). FAK-associated proteins have been reported to be involved in mediating a range of important cellular functions, including development, immune response, cardiovascular function, and maintenance of tissue integrity. FAK phosphorylation is essential for transporting the viral capsid to the nuclear pore (30, 31). After HSV-1 infection of the corneal epithelium, the FAK signaling pathway is activated, leading to increased secretion of matrix metalloproteinase-2 in the corneal tissue and accelerating the formation of corneal ulcers and necrotic lesions (30). FAK phosphorylation is essential for transporting the viral capsid to the nuclear pore (31).

To further confirm the activation of ECM-ITGA/ITGB-p-FAK signaling pathway, we examined the expression levels of p-FAK-related proteins after PRV infection using western blotting. The results showed that the protein levels of ECM, ITGA/ITGB, and p-FAK were significantly increased in the challenged group (Group A), whereas there was no difference between the immunization-challenged group (Group B), and a mock group (Group C). The results of western blotting and RT-qPCR were in agreement (Fig. 7). Therefore, we hypothesized that PRV promotes self-infection through activation of the ECM-ITGA/ITGB-p-FAK signaling pathway and that PRV XJ delgE/gI/TK could attenuate the intestinal damage caused by PRV by inhibiting the activation of this pathway.

## Data Availability

The data presented in the study are deposited in the NCBI SRA repository, accession number PRJNA1138528. The data are also included in the article/Supplementary Material. Further inquiries can be directed to the corresponding author.

## SUPPLEMENTAL MATERIAL

Figure S1 spectrum.01828-24-s0001.tif Ribosomal RNA peaks of all samples.

Table S1 spectrum.01828-24-s0002.xlsx GO enrichment analysis of differentially expressed genes in pairwise comparisons among three groups.

Table S2 spectrum.01828-24-s0003.xlsx Enrichment analysis of KEGG pathway differentially expressed genes in pairwise comparisons among three groups.

ASM does not own the copyrights to Supplemental Material that may be linked to, or accessed through, an article. The authors have granted ASM a non-exclusive, world-wide license to publish the Supplemental Material files. Please contact the corresponding author directly for reuse.
