# Supplementary figures and images for: Transcriptome analysis reveals that PRV XJ delgE/gI/TK protects against intestinal damage in nose-dropping-infected mice by regulating ECM-ITGA/ITGB-P-FAK
Source: Microbiol Spectr. 2024 Nov 29;13(1):e01828-24. doi: 10.1128/spectrum.01828-24 (PMC11705885; doi:10.1128/spectrum.01828-24)

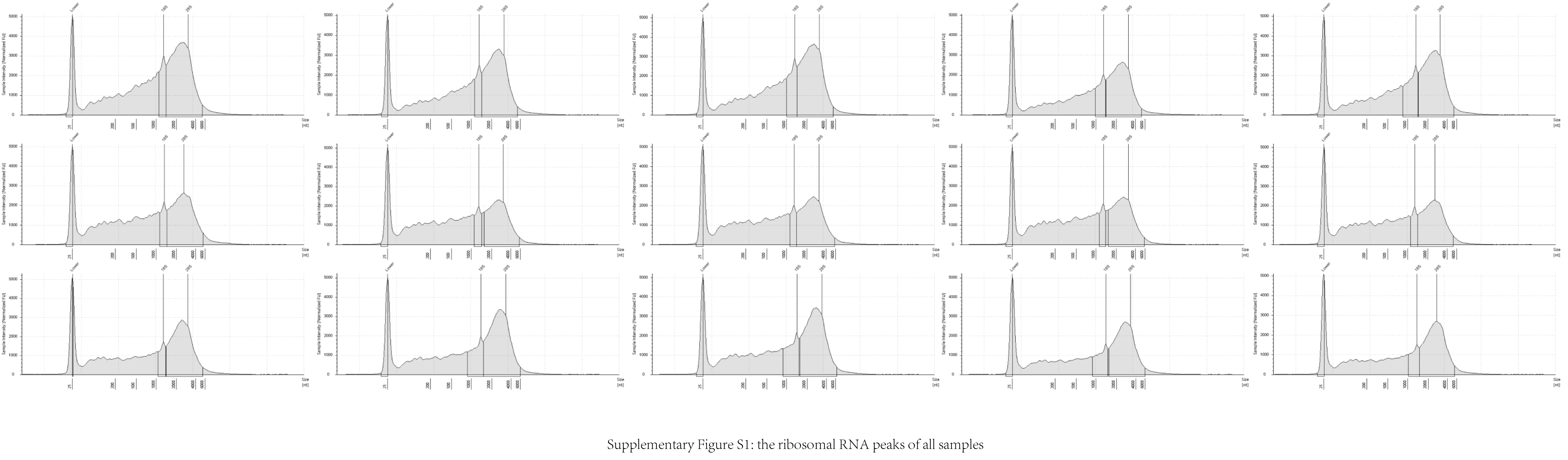

Supplement: Figure S1 — Ribosomal RNA peaks of all samples. [file spectrum.01828-24-s0001.tif]
